# Uncertainty in cardiac myofiber orientation and stiffnesses dominate the variability of left ventricle deformation response

**DOI:** 10.1002/cnm.3178

**Published:** 2019-01-21

**Authors:** Rocío Rodríguez‐Cantano, Joakim Sundnes, Marie E. Rognes

**Affiliations:** ^1^ Department of Numerical Analysis and Scientific Computing Simula Research Laboratory AS Bærum Norway; ^2^ Center for Cardiological Innovation Simula Research Laboratory Bærum Norway

**Keywords:** cardiac mechanics, Karhunen‐Loéve expansion, polynomial chaos, quasi‐Monte Carlo, sensitivity analysis, uncertainty quantification

## Abstract

Computational cardiac modelling is a mature area of biomedical computing and is currently evolving from a pure research tool to aiding in clinical decision making. Assessing the reliability of computational model predictions is a key factor for clinical use, and uncertainty quantification (UQ) and sensitivity analysis are important parts of such an assessment. In this study, we apply UQ in computational heart mechanics to study uncertainty both in material parameters characterizing global myocardial stiffness and in the local muscle fiber orientation that governs tissue anisotropy. The uncertainty analysis is performed using the polynomial chaos expansion (PCE) method, which is a nonintrusive meta‐modeling technique that surrogates the original computational model with a series of orthonormal polynomials over the random input parameter space. In addition, in order to study variability in the muscle fiber architecture, we model the uncertainty in orientation of the fiber field as an approximated random field using a truncated Karhunen‐Loéve expansion. The results from the UQ and sensitivity analysis identify clear differences in the impact of various material parameters on global output quantities. Furthermore, our analysis of random field variations in the fiber architecture demonstrate a substantial impact of fiber angle variations on the selected outputs, highlighting the need for accurate assignment of fiber orientation in computational heart mechanics models.

## 1 INTRODUCTION

Computational modelling of the heart is a powerful technique for detailed investigations of cardiac behavior and enables the study of mechanisms and processes that are not directly accessible by experimental methods. There is currently a drive towards adapting these computational models to individual patient data, to aid in the creation of individualized diagnosis, clinical decision support, and treatment planning.^1^, ^2^, ^3^, ^4^, ^5^, ^6^, ^7^ However, this model adaptation presents a number of challenges related to the lack of available data and the fact that measurable data, needed for patient‐specific model input parameters, are inherently subject to measurement uncertainties or intrinsic biological variability. For clinical use of models, it is therefore of crucial importance to quantify how these uncertainties propagate through the computational model to impact the output quantities of interest. Such assessment should be performed with uncertainty propagation and uncertainty quantification (UQ) techniques,^8^, ^9^ complemented by sensitivity analysis (SA) to identify the most significant input variables.^10^

In the particular case of cardiac ventricular mechanics studies, individualized model adaption involves image‐based construction of computational geometries as well as tuning of material parameters.^11^, ^12^, ^13^, ^14^, ^15^, ^16^, ^17^, ^18^ Since the mechanical properties of cardiac tissue are strongly anisotropic, the local material behavior typically depends both on a set of material parameters and on the local orientation of the cardiac muscle cells, typically referred to as the fiber and sheet orientation. The local tissue structure can be determined with diffusion tensor magnetic resonance imaging (DTMRI), but this technique is still limited to ex vivo experiments. Patient‐specific models have been created by projecting ex vivo DTMRI datasets onto patient‐specific geometries obtained from computed tomography (CT) or magnetic resonance imaging (MRI).^11^, ^19^, ^20^, ^21^, ^22^ However, even in the in vitro case, the accuracy of DTMRI is ±10°.^23^, ^24^, ^25^ While this accuracy may be sufficient in the context of computational cardiac electrophysiology,^26^ local variations of this order have been shown to introduce sizeable variations in myofiber stresses.^27^

Rule‐based or atlas‐based methods represent a convenient alternative for assigning fiber and sheet orientation in patient‐specific models. For instance, the Laplace‐Dirichlet rule‐based (LDRB) algorithm^16^ is based on atlas data and assigns a generic tissue architecture to image‐based patient‐specific geometries. This method obviously neglects potential individual variations in tissue structure but provides a reasonable averaged fiber/sheet orientation. Lombaert et al^20^ built the first statistical atlas of the cardiac fiber architecture using human datasets (10 ex vivo hearts imaged with DTMRI), providing the spatial distribution of fiber angles with their variability within the healthy population. Their results showed that the helix angle of the fibers varies globally from −41° (±26°) on the epicardium to −66° (±15°) on the endocardium. The reported variability includes both true variability of the fiber structure and errors due to acquisition and image registration. Similarly, Molléro et al^28^ estimated and represented the uncertainty of cardiac fiber architecture originating from the lack of data for a given patient using the mean and principal modes of variations among a given population of healthy hearts.

In spite of the potential impact for clinical use of the models, there are relatively few examples of proper UQ and SA for mechanical models of the heart. Osnes and Sundnes^29^ and Hurtado et al^30^ studied the impact of uncertainty in material parameters, while Puijmert et al^31^ investigated the sensitivity of a cardiac mechanics model to changes in myofiber orientation over an average angle of about 8°. An increase in total pump work of 11% to 19*%* was found in three different geometries, revealing that implementing an accurate fiber field is important for achieving the correct model output. Sensitivity of cardiac models to the myofiber orientation was also highlighted in previous studies.^18^, ^32^, ^33^

One explanation for limited use of UQ and SA in cardiac modeling is the computational expense of the involved models. A popular statistical approach is the Monte Carlo (MC) method, but this method typically requires a large number of model evaluations for converged results. If the base model is a realistic computational model of cardiac mechanics, the resulting computational cost will be substantial. Techniques such as the quasi‐Monte Carlo (QMC)^34^, ^35^ and the multilevel Monte Carlo (MLMC)^36^ methods can significantly improve the MC convergence rate, but their application may be limited and technically complex. Recently, alternative approaches, such as the use of surrogate models^37^ to mimic the behavior of the full model while being inexpensive to evaluate, have been of particular interest. One such technique is the *polynomial chaos expansion* method (PCE),^38^, ^39^ which has previously been used in UQ analysis of cardiac mechanics and electrophysiology.^29^, ^40^

The purpose of the present work is to present a PCE‐based method for UQ in cardiac mechanics models and to perform an initial UQ and SA study including both global myocardial material properties and local variability of the microstructure orientation. The study of global material parameters is similar to the UQ analysis in Osnes and Sundnes,^29^ but using a more realistic computational model and including a detailed SA of key input‐ and output variables. The UQ considering local variations in microstructure orientation is, to our knowledge, the first of its kind. In this case, the input was treated as a random field, and modeled as a truncated Karhunen‐Loève expansion (KLE)^41^ in order to reduce the dimensionality of the random field representation. The former is used as a basis to build a reduced‐dimensionality representation of the random field, essential to manage UQ analysis in extremely high‐dimensional problems. Although the fiber arrangement exhibit a typical gross architecture, as we mention above, there are local and individual variations through the ventricular wall, as well as uncertainty derived from noisy measurements that may affect the global mechanical properties of the model. The results give insight into the applicability of the truncated KLE method for representing noisy fiber architecture fields, and to the impact of such variations on global response quantities.

## 2 MODELS AND METHODS

The overarching objective of this paper is to illustrate and evaluate the impact of input data uncertainty on the mechanical response of the heart. We introduce the forward model for the mechanical behavior of the left ventricle and its numerical approximation in Section 2.1 below and describe our UQ techniques subsequently in Section 2.2

### 2.1 Cardiac ventricular forward model

#### 2.1.1 Governing equations

Let $D\subset R^{3}$ be the computational domain representing the left ventricle. We consider the quasi‐static and pressure‐loaded mechanical equilibrium problem over this domain: find the displacement $u:D\to R^{3}$ such that

(1) −∇·(FS)=0inD,

where ***F*** is the deformation gradient, ie, ***F***=∇***u***+***I***, and ***S*** is the second Piola‐Kirchhoff stress tensor. Boundary conditions for (1) are described below.

We assume that the material is hyperelastic and therefore that the Piola‐Kirchhoff stress tensor ***S*** is the derivative of a strain energy density Ψ  =  Ψ(***E***) with respect to the Green‐Lagrange strain tensor ***E***, defined as

(2) $$E=\frac{1}{2}\left(F^{T}F-I\right).$$

In particular, we consider a transversely isotropic, hyperelastic, and almost incompressible material and apply the widely used constitutive model of Guccione et al.^42^ This model is defined relative to three mutually orthogonal vector fields: a fiber field $f:D\to R^{3}$, a fiber sheet field $s:D\to R^{3}$, and a sheet normal field $n:D\to R^{3}$. The strain energy density is then defined as

(3) $$\Psi (E)=\frac{1}{2}C(e^{W}-1)+K(J\ln J-J+1)$$

with

(4) $$W=b_{ff}E_{ff}^{2}+b_{xx}(E_{ss}^{2}+E_{nn}^{2}+E_{sn}^{2}+E_{ns}^{2})+b_{fx}(E_{fn}^{2}+E_{nf}^{2}+E_{fs}^{2}+E_{sf}^{2}).$$

Here, *E*_*ij*_ are components of the Green‐Lagrange strain tensor in the local fiber (*f*), fiber sheet (*s*), and sheet normal (*n*) axis, ie, *E*_*ij*_  =  *j*·***E*** *i* for directions *f*, *s*, and *n*. Additionally, *J* is the determinant of the deformation gradient, and *C*, *K*, *b*_*ff*_, *b*_*xx*_ and *b*_*fx*_ are material parameters. In particular, *b*_*ff*_ and *b*_*xx*_ are parameters governing the material stiffness in the fiber and cross‐fiber directions, respectively, *b*_*fx*_ represents the shear stiffness in planes parallel to the fibers, *K* is the incompressibility factor of the myocardial tissue, and *C* enters as a multiplicative factor in the strain energy function.

#### 2.1.2 Geometry, mesh, and fiber orientations

**Figure 1:**
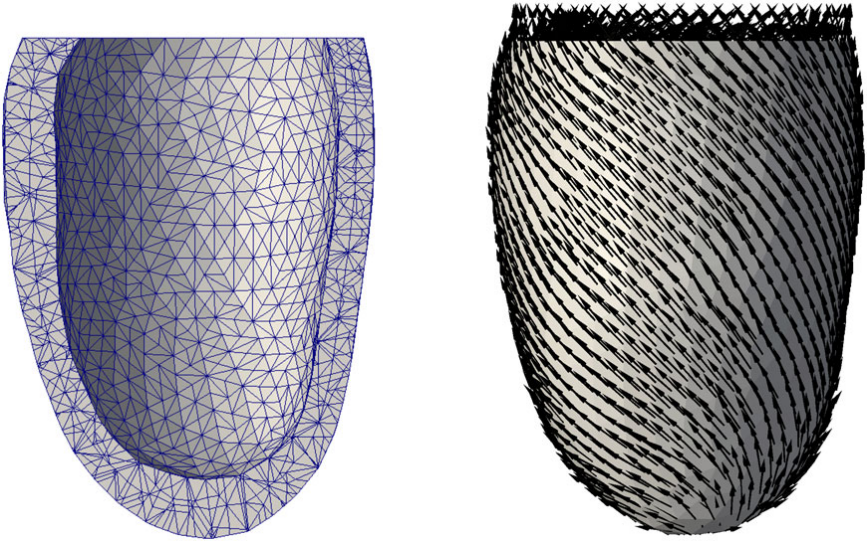
Left: Computational mesh of a human left ventricle wall. Right: Baseline fiber orientation field f over the computational mesh

A computational mesh of the domain *D* was generated from an echocardiographic image of a left ventricle at the beginning of atrial systole using the EchoPac software package (GE Healthcare Vingmed) and Gmsh. We constructed a flat ventricular base by cutting the geometry with a plane fit to the points on the base. Although the images correspond to a loaded state of the ventricle, with a non‐zero cavity pressure, we used this geometry as the unloaded reference state in our model. While this choice would not be appropriate for creating a patient‐specific mechanics model, it was deemed sufficient for testing the UQ methods and the general impact of uncertainty in material parameters. The resulting tetrahedral mesh is shown in Figure 1 (left), counting 18 112 tetrahedra. Quadratic basis functions were used in the FE discretization, with 36 223 nodes in total.

As note above, the model (3) assumes the availability of local coordinate systems *f*,*s*,*n* aligned with the local orientation of muscle fibers. While the fiber orientation is not generally possible to measure in vivo, it is known that the fiber axes follow a helical pathway as illustrated in Figure 1 (right) with a counterclockwise rotation of the helix angle from epicardium to endocardium.^43^ In view of this, we applied a Laplace‐Dirichlet rule‐based (LDRB) algorithm^16^ to generate realistic fiber‐, fiber sheet‐, and sheet normal orientation fields in our ventricular model. The LDRB method defines two main angles to describe the local tissue structure. The fiber angle *α* defines the orientation of the longitudinal fiber direction relative to the circumferential direction, while *β* is the angle between the transverse fiber direction (in the plane of the sheets) and the outward transmural axis of the heart.

Input parameters to the model are the values of these angles on the endocardial and epicardial surfaces, respectively: *α*_*endo*_, *α*_*epi*_, *β*_*endo*_, and *β*_*epi*_. Pointing ahead, in the present study, we will both consider these input angles as random variables, as previously done in Osnes and Sundnes,^29^ and also apply a Karhunen‐Loéve expansion (cf. Section 2.2.4) to study the impact of random variations in the full fiber field.

#### 2.1.3 Boundary conditions

Following, eg, Balaban et al^44^ to constrain the displacement at the base of the left ventricle boundary, we applied a Robin boundary condition with a spring constant of 1 kPa. Moreover, we let the base of the left ventricle be clamped (zero displacement) in the longitudinal direction. At the endocardium (inner) surface, we applied a pressure of 2 kPa, corresponding to the end‐diastolic pressure, as a normal stress boundary condition. At the epicardium (outer) surface, we assumed zero normal stress.

#### 2.1.4 Numerical discretization

To solve (1) with the previously described boundary conditions, we considered a finite element discretization. The fiber‐, fiber sheet‐, and sheet normal orientation fields were interpolated onto continuous piecewise linear vector fields defined relative to the computational mesh, and we similarly approximated the displacement field using continuous piecewise linear vector fields. The nonlinear systems of equations were solved using Newton method, and the resulting linear equations were solved using a direct method. The endocardial pressure was applied incrementally to improve the nonlinear convergence.

### 2.2 Uncertainty quantification

For brevity, in the presentation of the UQ techniques, we will denote the finite element discretization of the forward model described by (1) and associated boundary conditions by 𝒴. In general, this forward model can be viewed as a function, over the space ***x***∈*D*, mapping a set of input parameters ***η*** to output values *Y*:

(5) Y=𝒴(x,η).

The mapping 𝒴 is deterministic, so that when evaluated on the same *d* input parameters ***η***=(*η*_1_,…,*η*_*d*_), it yields the same specific output values *Y*.

We will consider both the case where each *η*_*i*_ represents a (single) random variable and the case where some *η*_*i*_ represent a random field. Concretely, ***η*** will represent ventricular material parameters such as *C*,*K*,*b*_*ff*_,*b*_*fx*_,*b*_*xx*_ and the input parameters of the fiber field model *α*_*endo*_,*α*_*epi*_,*β*_*endo*_,*β*_*epi*_, or variables associated with the uncertainty in the orientation field *f*.

A UQ analysis evaluates the impact in output *Y* that results from the uncertainty in the parameters ***η***, assuming a known joint probability distribution *p*_***η***_ associated with the input vector ***η***. The most popular technique for UQ analysis is MC simulation, which involves the use of a sampling method to draw a set of samples from the parameter space. Relevant statistics of the output *Y* is obtained by evaluating the deterministic model (5) on the sampling set. Although simple and widely applicable, the MC technique converges slowly and typically requires a large number of evaluations of the forward model 𝒴. In our case, each evaluation involves solving a nonlinear finite element model, leading to a substantial computational cost. We have therefore considered alternative techniques to reduce the required number of 𝒴 evaluations.

#### 2.2.1 Polynomial chaos expansion

The polynomial chaos expansion (PCE) method^38^ expands the uncertain model outputs in a suitable series, which mimics the behavior of the forward model (5) but is much cheaper to evaluate. This series expansion can then be used to perform cheap UQ and SA, using sampling techniques such as the QMC method.^34^, ^35^ In PCE, evaluations of the forward model (5) are required to build the series expansion, but the number of required model evaluations is normally lower than for standard sampling methods.

Assuming that the output of interest from (5) is a smooth function of *d* random input parameters ***η***=(*η*_1_,…,*η*_*d*_), the PCE approximates *Y* as a function of ***η*** by a truncated polynomial expansion as follows^45^:

(6) $$Y(x,\eta )\approx Ŷ(x,\eta )=\sum_{i=1}^{M}c_{i}(x)\Phi_{i}(\eta ).$$

Here, {Φ_*i*_} is a given multivariate orthogonal polynomial basis for ***η***, *c*_*i*_(***x***) are the coefficients that quantify the dependence of the model output on the parameters ***η***, and *M* is the total number of expansion terms. This number is determined by the dimension *d* of the random vector ***η*** and the highest order *N* of the polynomials {Φ_*i*_}, more precisely *M*  =  (*N* + *d*)! (*N*! *d*!)^−1^. This number grows rapidly with *N* and *d*, which may render the approach computationally unaffordable for large numbers of uncertain parameters, and has motivated adaptive methods for constructing sparse PCE bases.^46^, ^47^, ^48^, ^49^, ^50^ However, for the number of unknown parameters considered here, we have found a fixed basis of Hermite polynomials to give sufficiently accurate results. The Hermite polynomials have been shown to give optimal convergence for normal distributions,^51^ and we use a combination of normal and log‐normal distributions for our input parameters. The deterministic functions *c*_*i*_(***x***) may be computed by the point collocation method.^52^ Within this technique, the unknown coefficients of the expansion are estimated by equating model outputs and the corresponding polynomial chaos expansion at a set of collocation points in the parameter space. For each output of the model, a set of linear equations is formed with the coefficients as the unknowns:

(7) $$\left(\begin{array}{ccc} \Phi_{1}(q_{1})\; & \text{⋯} & \Phi_{M}(q_{1})\\ \vdots & \ddots & \vdots \\ \Phi_{1}(q_{N_{s}}) & \text{⋯} & \Phi_{M}(q_{N_{s}}) \end{array}\right)\left(\begin{array}{c} c_{1}(\cdot )\\ \vdots \\ c_{M}(\cdot ) \end{array}\right)=\left(\begin{array}{c} 𝒴(\cdot ,q_{1})\\ \vdots \\ 𝒴(\cdot ,q_{N_{s}}) \end{array}\right)$$

The collocation points $\left\{q_{1},\text{⋯}q_{N_{s}}\right\}$ must be chosen in a way so that the matrix (7) is well conditioned.^53^ This requirement allows for the use of conventional QMC sampling methods^54^ to select a number of collocation points equal or greater than the number of unknown coefficients *c*_*i*_(***x***).^52^

Once the coefficients are determined and Ŷ(x,η) is built, the last step in the PCE method for UQ is to propagate the uncertainties through the simulator in order to estimate statistics of the response quantities. This last step is performed by MC simulations, in which the model solver of (5) is substituted by the surrogate Ŷ as a cheaper alternative. It is important to note that for PCE, the convergence depends on both the maximal order *N* of the polynomials {Φ_*i*_} and the number of collocation points *N*_*s*_ selected to build Ŷ. We return to this point in Section 3. Typical statistical response quantities include expected value (*μ*), standard deviation (*σ*), prediction intervals, and coefficient of variation, in order to characterize the probability density function (pdf) corresponding to each output quantity of interest.^55^

#### 2.2.2 Quasi‐Monte Carlo

We have also applied quasi‐Monte Carlo (QMC)^34^, ^35^ simulations with Halton low‐discrepancy sampling sequences^56^ to verify and validate the results obtained by the PCE methods.

#### 2.2.3 Sobol sensitivity indices

In addition to computing statistical properties of the output probabilities, we perform SA^10^, ^57^, ^58^ to quantify the contribution of a particular input *η*_*i*_, and of specific parameter interactions, to the output variance. This analysis may be useful for model personalization, for which *input fixing* (identify non‐influential parameters to fix them within their uncertainty domain) and *input prioritization* (determination of which factor(s), once fixed to its true value, leads on average to the greatest reduction in the variance of an output) are important goals. In this study, we compute the total ( $S_{i}^{T}$) and the main (*S*_*i*_) variance‐based Sobol sensitivity indices,^59^ which can be used for *input fixing* and *input prioritization*, respectively.

Specifically, the main sensitivity index *S*_*i*_ is the proportion of the total variance V of *Y* that is expected to be reduced if *η*_*i*_ was fixed on its unknown true value. It can be computed according to^60^

(8) $$S_{i}=\frac{V[E[Y|\eta_{i}]]}{V[Y]},$$

where the index *i* varies from 1 to the number of random inputs *d* and E is the expected value of the output quantity in question *Y*. Furthermore, the total sensitivity index $S_{i}^{T}$, which represents the total variance due to both the direct effect and all input interactions of *η*_*i*_, is given by^60^

(9) $$S_{i}^{T}=\frac{V[Y]-V[E[Y|\eta_{i*}]]}{V[Y]},$$

in which ***η***_*i*∗_ contains all uncertain inputs except *η*_*i*_.

#### 2.2.4 Karhunen‐Loève expansion

One of the key goals of this paper is to quantify and evaluate the impact of uncertainty originating from the variability of the myofiber orientation field *f*; cf (4). As a statistical model for an input that addresses variability as function of space, it must be described by a random field variable. In particular, in the following, we will consider a random myofiber orientation field as the sum of a random field perturbation and a fiber field generated by the LDRB method of Bayer et al.^16^

(10) $$f(x,\theta )=f_{LDRB}(x)+F(x,\theta ),\;x\in D,$$

where *θ*  ∈  Ω denotes the dependency of *f* on some random property. To represent the random field perturbation, we make use of a truncated Karhunen‐Loéve expansion. Any second‐order random field *F*(***x***,*θ*) defined over *D* × Ω, with covariance function *C* and expected value $\bar{F}$, can be represented by the Karhunen‐Loéve expansion,^41^, ^61^ also known as the proper orthogonal decomposition, as the following infinite linear combination of orthogonal functions:

(11) $$F(x,\theta )=\bar{F}(x)+\sum_{k=1}^{\infty }\eta_{k}(\theta ){\sqrt{\lambda_{k}}\phi }_{k}(x).$$

In (11), $\bar{F}(x)$ is the expected value of the stochastic field at ***x***, {*η*_*k*_(*θ*)} represents a set of uncorrelated random variables (if *F*(***x***,*θ*) is assumed to be Gaussian then {*η*_*k*_(*θ*)} are also independent), and {*λ*_*k*_, ϕ_*k*_(***x***)} are eigenvalues and eigenfunction pairs of the homogeneous Fredholm integral equation over *D*:

(12) $$\int_{D}C(y,x){\;\phi }_{i}(y)\;dy={\lambda {}_{i}\phi }_{i}(x),$$

using the covariance function *C*(***y***,***x***) as kernel.^61^

In practice, the infinite series in (11) may be truncated after the terms corresponding to the highest *n*_KL_ eigenvalues {*λ*_*k*_}:

(13) $$F(x,\theta )\approx \tilde{F}(x,\theta )=\bar{F}(x)+\sum_{k=1}^{n_{\text{KL}}}\eta_{k}(\theta ){\sqrt{\lambda_{k}}\phi }_{k}(x).$$

The number of terms *n*_KL_ depends on the decay of eigenvalues, which in turn depends on the smoothness of the covariance function *C*. If the eigenvalues {*λ*_*k*_} decay sufficiently fast and *n*_KL_ is large enough, $\tilde{F}$ provides a suitable approximation of *F*.

In this study, as we consider a random field perturbation to the myofiber orientations, we assume $\bar{F}(x)=0$, without loss of generality. Moreover, we have chosen the squared exponential covariance structure^62^ as the covariance function;

(14) $$C(x,y)=\sigma_{\text{KL}}^{2}\exp(-\frac{|x-y{|}^{2}}{2l^{2}})\;\forall \;x,y\in D.$$

Here, $\sigma_{\text{KL}}^{2}$ is the field variance controlling the typical amplitude of the random field, and *l* is the correlation length that defines the typical length‐scale over which the field exhibits significant correlations. Considering the lack of experimental data from which to estimate the spatial uncertainty associated to the myofiber orientation field, we consider this choice of correlation function to be a sensible starting point for study. Finally, in this study, the approach of truncation has been to examine the decay of the eigenvalues {*λ*_*k*_} in (11) and keep the first *n*_KL_ eigenvalues {ϕ_*k*_(***x***)} so that the contributions from the remaining eigenvalues are negligible.

This reduction of dimensionality of the stochastic space, from infinite to *n*_KL_, provides a parametric representation of the random field *F*(***x***,*θ*) through *n*_KL_ random variables. The uncertainty of the fiber field now stems from the vector of parameters ***η***=(*η*_1_,…,*η*_KL_), with {*η*_*k*_} the uncorrelated random variables defined in (11). Standard uncertainty propagation methods, like MC or PCE, can be used then to predict the influence of the variability of the myofiber orientation (10) on our model. As an error measure for the random field truncation (13), we have used the error variance introduced by Betz et al.^63^ In particular, *n*_KL_ has been selected ensuring that in more than the 92*%* of the discretized points ***x***, the error variance is lower than 0.05. In our experiments, *n*_KL_ range from 4 to 16 depending on the correlation length *l* in (14).

#### 2.2.5 Computing Karhunen‐Loéve approximation

Analytical solutions of the eigenvalue problem (12) rarely exist, so in general, it has to be solved numerically.^64^, ^65^ For this purpose, we consider the weak formulation (Galerkin projection) of the system of Equation 12 on a discretization of the domain *D*. In particular, assume that we have a mesh $T_{h}$ of the fixed domain *D* with vertices (nodes) *x*_1_…,*x*_*n*_. Take a continuous piecewise linear basis {*v*_1_,…,*v*_*n*_} defined relative to this mesh and consider the generalized eigenvalue problem^66^: find ϕ_*k*_ and *λ*_*k*_ such that

(15) $${T\phi }_{k}=\lambda_{k}{M\phi }_{k},$$

where *M* is the mass matrix:

(16) $$M_{ij}=\int_{D}v_{i}(x)v_{j}(x)\;dx,$$

and

(17) T=MQM

with *Q*_*ij*_  =  *C*(***x***_*i*_,***x***_*j*_) the covariance matrix that emerges from the discrete representation of the random field with covariance kernel *C*.

It is important to note that while the mass matrix *M* is symmetric positive definite and may be sparse, *T* is symmetric positive semidefinite and dense. Since *Q* is dense, we applied a data sparse technique to store it with the hierarchical matrix ( H‐matrix) format.^66^, ^67^ Consequently, the computational cost of matrix‐vector products involving *Q* is reduced from $O(n^{2})$ to O(nlogn), with *n* the number of discretization points. The H‐matrix technique is a hierarchical division of a given matrix into rectangular blocks and further approximation of these blocks by low‐rank matrices.^68^, ^69^, ^70^ In order to compute the low‐rank approximations, the adaptive cross‐approximation (ACA) algorithm^71^ was employed.

#### 2.2.6 Statistical properties of random input quantities

In this study, we introduce two different models of uncertainty. First, we consider the material stiffnesses *b*_*ff*_,*b*_*xx*_,*b*_*fx*_, the incompressibility parameter *K* and the weighting factor *C* as uncertain (random) variables of prescribed probability distributions. The mean values of these parameters were taken from Usyk et al,^72^ with statistical properties chosen as in Osnes and Sundnes.^29^ Moreover, we similarly treat randomness in fiber orientations as a direct function of the random input variables *α*_*endo*_, *α*_*epi*_, *β*_*endo*_, and *β*_*epi*_ to the LDRB algorithm. For these variables, we have assumed a normal distribution with expected values similar to the default values from Bayer et al^16^ and a coefficient of variation equal to 0.15. The prescribed distributions, expected values, and standard deviations are listed in Table 1, and we refer to this case as model A. All parameters are treated as independent.

**Table 1:** Statistical properties of the input parameters in model A: probability distribution ( $\rho_{\eta_{i}}$), expected value ( $\mu_{\eta_{i}}$), and standard deviation ( $\sigma_{\eta_{i}}$)

Parameter	Unit	$\rho_{\eta_{i}}$	$\mu_{\eta_{i}}$	$\sigma_{\eta_{i}}$
*b*_*ff*_		Normal	6.6	0.99
*b*_*xx*_		Normal	4.0	0.6
*b*_*fx*_		Normal	2.6	0.39
*K*	kPa	Log‐normal	10.0	1.5
*C*	kPa	Log‐normal	1.1	0.165
*α*_*endo*_	degree	Normal	50.0	7.5
*α*_*epi*_	degree	Normal	−50.0	7.5
*β*_*endo*_	degree	Normal	−65.0	9.75
*β*_*epi*_	degree	Normal	25.0	3.75

In the second model (model B), we introduce uncertainty in the fiber orientation field only by adding a Gaussian random field to the fiber architecture generated by the LDRB algorithm. We thus introduce a nonuniform perturbation in angle orientation of every fiber axis over the computational geometry. The random perturbation field is approximated via the truncated Karhunen‐Loéve expansion as described in Section 2.2.4. The properties of the random field depend strongly on the selected correlation length. We have considered three different correlation lengths, *l*  =  3,5, or 10 cm, and two different standard deviations, *σ*_KL_  =  0.1 and 0.5 radians, respectively. In this second model (model B), five material parameters *C*,*K*,*b*_*ff*_,*b*_*fx*_,*b*_*xx*_ and the angles *α*_*endo*_,*α*_*epi*_,*β*_*endo*_,*β*_*epi*_ are kept fixed at their mean value given by Table 1.

**Figure 2:**
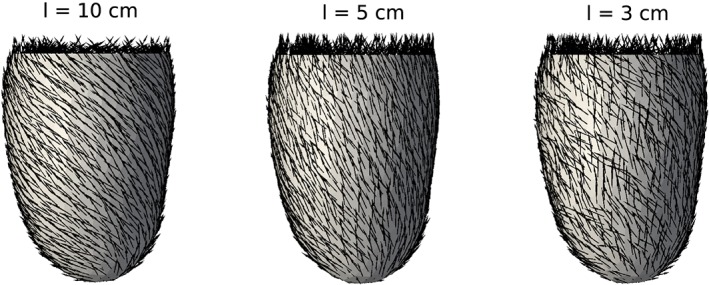
Samples of random fiber orientation fields; f(**x**,θ) in (10), with the noise represented by the Karhunen‐Loéve decomposition (Model B). The figure shows Gaussian random fields, all with a standard deviation σ_KL_  =  0.5 radians, and correlation lengths l  =  10 cm (left), l  =  5 cm (middle), and l  =  3 cm (right)

Three samples of the different (total) random fiber orientation fields, *f*(***x***,*θ*) in (10) assuming a standard deviation of 0.5 radians, are illustrated in Figure 2. Note that short correlation lengths in the random field generates strong fluctuations in the fiber architecture, while a higher value of *l* implies that the random field approaches a random variable (ie, constant over the computational domain). For long correlation lengths, the generated fields are similar to those generated by treating input parameters *α*_*endo*_, *α*_*epi*_, *β*_*endo*_, and *β*_*epi*_ as stochastic, making model B very similar to model A described above. However, for short correlation lengths, the two models are qualitatively very different, as model B creates far less structured and organized fiber distributions. The required number of terms *n*_KL_ in the Karhunen‐Loéve decomposition (13) varies from 4, 9, and 16 with decreasing *l*, so the more correlated the orientation field, the smaller the number of terms necessary to retain its essential information in the truncated Karhunen‐Loéve expansion.

#### 2.2.7 Quantities of interest

As quantities of interest (or target values) we have chosen global, observable quantities: the volume of the inner cavity *Q*_*c*_, the lengthening of the apex *Q*_*l*_ (difference between epicardial and endocardial axial length), the change in wall thickness *Q*_*t*_ (difference between outer and inner radius at base), and the total wall volume *Q*_*v*_. These quantities of interest were chosen to have a compact set of scalar output quantities that characterize the global ventricular deformation. Being global measures, these output quantities are strongly intercorrelated, and we therefore expect to see some similarities and correlation in the parameter sensitivities.

The reference values of these quantities of interest (corresponding to the reference configuration of the ventricular domain at zero endocardial pressure) are given in Table 2.

**Table 2:** Quantities of interest corresponding to the reference configuration of the ventricular domain

Quantity of interest (Unit)	Reference value
Inner cavity volume *Q*_*c*_ (10^2^ × cm^3^)	1.70
Apex lengthening *Q*_*l*_ (cm)	1.11
Wall thickness *Q*_*t*_ (10^−1^ cm)	6.99
Wall volume *Q*_*v*_ (10^2^ × cm^3^)	1.26

### 2.3 Implementation

We used the Python interface to the FEniCS finite element software^73^, ^74^ to implement the forward model described in Section 2.1. The UQ analysis was performed using the ChaosPy toolbox,^75^ using the FEniCS forward solver as a black box model. We also used FEniCS to assemble the matrices *T* and *M* in (15). Finally, the dominant eigenmodes of the eigenvalue problem (15) (approximating the eigenmodes of (12)) were obtained using ARPACK accessed via SciPy.^76^

## 3 RESULTS

The main focus of this work is to quantify the impact of uncertainty in local myofiber architecture on representative global response quantities of interest. Prior to the main study focusing on models A and B as described above, we present results from the calibration of the surrogate PCE models.

### 3.1 Surrogate model calibration and validation of statistical outputs

The performance of the PCE model depends on the choice of polynomial order *N*, and the number of sampling points *N*_*s*_ used to fit the surrogate model to the finite element model. Once both the maximum polynomial order and the number of uncertain parameters (*d*) are set, the number of expansion terms or unknown coefficients, *M*, can be calculated as explained in Section 2. For each of the experiments considered below, we conducted a series of numerical experiments to choose suitable values for *N* and *N*_*s*_. Specifically, we experimented with polynomial order *N* from 1 to 3, and number of sampling points *N*_*s*_ set to 2*M*,3*M*, and 4*M* and compared the results in terms of root mean‐square error (RMSE) between the surrogate and the forward (finite element) model outputs for a new/different set of points in the parameter space. We found that a polynomial order *N*  =  2 and number of sampling points *N*_*s*_  =  2*M* gave sufficient accuracy for our cases. The results are summarized in Tables 3 and 4. Table 3 shows the number of samples needed for the PCE method and QMC in model A, where all parameters are treated as scalar global quantities. All the PCE results are for polynomial order *N*  =  2, while the number of QMC samples was chosen to obtain converged results for each quantity of interest. Table 4 shows similar results for model B, where the fiber orientations are modeled as stochastic fields, for three different choices of the correlation length *l*.

**Table 3:** Summary of calibration and validation results for model A^a^

Quantity	*N*	*N*_*s*_	QMC	RMSE
Inner volume (10^2^ × cm^3^)	110	250	2.1 × 10^−2^
Lengthening (cm)	110	250	2.7 × 10^−4^
Wall thickness (10^−1^cm^3^)	110	450	2.7 × 10^−4^
Wall volume (10 × cm^3^)	110	450	1.6 × 10^−5^

a Column *N*_*s*_ is the number of samples (forward solves) needed to construct the PCE model and QMC the number of samples needed for convergence of the QMC method. The rightmost column shows the error of the PCE model, measured as the difference between the PCE model and the forward finite element model.

**Table 4:** Summary of validation results for model B^a^

*l*	Quantity	*N*_*s*_	QMC	RMSE
10	Inner volume (10^2^ × cm^3^)	30	180	3.4/0.02 ×10^−3^
	Lengthening (cm)	30	180	7.4/2.8 ×10^−5^
	Wall thickness (10^−1^ × cm)	30	250	1.94/0.001 ×10^−4^
	Wall volume (10 × cm^3^)	30	250	11/0.38 ×10^−5^
5	Inner volume (10^2^ × cm^3^)	110	250	1.1/0.01 ×10^−3^
	Lengthening (cm)	110	250	7.1/0.9 ×10^−5^
	Wall thickness (10^−1^ × cm)	110	450	1.29/0.0008 ×10^−4^
	Wall volume (10 × cm^3^)	110	450	6/0.23 ×10^−5^
3	Inner volume (10^2^ × cm^3^)	306	350	3.0/0.03 ×10^−3^
	Lengthening (cm)	306	350	1.5/0.6 ×10^−4^
	Wall thickness (10^−1^ × cm)	306	650	0.4/0.04 ×10^−4^
	Wall volume (10 × cm^3^)	306	650	0.1/0.4 ×10^−5^

a The leftmost column is the correlation length (*l*) used in the stochastic fields, while columns marked *N*_*s*_ and QMC are the number of samples (forward solves) used in the PCE model and QMC, respectively. The errors in the rightmost columns are reported for two choices of standard deviations in the stochastic fields, *σ*_*KL*_  =  0.5/0.1 radians, respectively.

Moreover, an extra convergence test has been performed comparing the standard deviation of every response quantity obtained via this validated single surrogate model with the same magnitude extracted from a QMC simulation through the Halton low‐discrepancy sampling sequence. The results are included in Tables 5, 6, 7. Overall, the nonintrusive PCE method was able to successfully generate a surrogate model for each quantity of interest specified in Table 2.

**Table 5:** Model A: statistical properties of the quantities of interest probability densities: expected value (μ), standard deviation (σ), coefficient of variation (cov = σ/μ), and prediction interval (PI_95_) via PCE^a^

Quantity	*μ*	*σ* (QMC)	cov	PI_95_
Inner volume (10^2^ × cm^3^)	3.81	0.30 (0.30)	0.08	[3.23, 4.39]
Lengthening (cm^3^)	0.83	0.05 (0.05)	0.06	[0.73, 0.93]
Wall thickness (10^−1^cm^3^)	4.79	0.28 (0.27)	0.06	[4.24, 5.34]
Wall volume (10 × cm^3^)	9.83	0.43 (0.42)	0.04	[8.90, 10.7]

a Standard deviation extracted from QMC simulations is also included (QMC).

**Table 6:** Model B: statistical properties of the output quantities distributions: expected value (μ), standard deviation (σ), coefficient of variation (cov = σ/μ), and prediction interval (PI_95_)^a^

*l* (cm)	Quantity	*μ*	*σ* (QMC)	cov (*σ*/*μ*)	PI_95_
10	Inner volume (10^2^ × cm^3^)	3.97	0.06 (0.06)	0.01	[2.79, 5.15]
	Lengthening (cm)	0.82	0.03 (0.03)	0.03	[0.76, 0.89]
	Wall thickness (10^−1^ × cm)	4.75	0.03 (0.03)	0.006	[4.69, 4.81]
	Wall volume (10 × cm^3^)	9.84	0.08 (0.09)	0.008	[9.68, 9.99]
5	Inner volume (10^2^ × cm^3^)	3.97	0.05 (0.05)	0.01	[3.87, 4.07]
	Lengthening (cm)	0.82	0.02 (0.02)	0.02	[0.78, 0.86]
	Wall thickness (10^−1^ × cm)	4.75	0.02 (0.02)	0.004	[4.71, 4.79]
	Wall volume (10 × cm^3^)	9.84	0.07 (0.07)	0.007	[9.70, 9.98]
3	Inner volume (10^2^ × cm^3^)	3.97	0.04 (0.04)	0.01	[3.89, 4.05]
	Lengthening (cm)	0.82	0.02 (0.02)	0.02	[0.78, 0.86]
	Wall thickness (10^−1^ × cm)	4.75	0.02 (0.02)	0.004	[4.71, 4.79]
	Wall volume (10 × cm^3^)	9.84	0.05 (0.05)	0.005	[9.74, 9.94]

a Gaussian random fields with a standard deviation *σ*_KL_  =  0.1 radians and correlation length *l* equals to 3, 5, and 10 cm. Standard deviation extracted from QMC simulations is also included (QMC).

**Table 7:** Model B: statistical properties of the output quantities distributions: expected value (μ), standard deviation (σ), coefficient of variation (cov = σ/μ), and prediction interval (PI_95_)^a^

*l* (cm)	Quantity	*μ*	*σ* (QMC)	cov (*σ*/*μ*)	PI_95_
10	Inner volume (10^2^ × cm^3^)	4.03	0.18 (0.17)	0.04	[3.70, 4.36]
	Lengthening (cm)	0.78	0.06 (0.06)	0.08	[0.66, 0.90]
	Wall thickness (10^−1^ × cm)	4.70	0.05 (0.06)	0.01	[4.60, 4.82]
	Wall volume (10 × cm^3^)	9.66	0.18 (0.17)	0.02	[9.31, 10.0]
5	Inner volume (10^2^ × cm^3^)	4.15	0.18 (0.17)	0.04	[3.80, 4.50]
	Lengthening (cm)	0.77	0.05 (0.05)	0.07	[0.67, 0.87]
	Wall thickness (10^−1^ × cm)	4.64	0.09 (0.09)	0.02	[4.47, 4.81]
	Wall volume (10 × cm^3^)	9.44	0.21 (0.21)	0.02	[9.04, 9.84]
3	Inner volume (10^2^ × cm^3^)	4.24	0.18 (0.18)	0.04	[3.89, 4.59]
	Lengthening (cm)	0.76	0.05 (0.05)	0.07	[0.66, 0.86]
	Wall thickness (10^−1^ × cm)	4.60	0.06 (0.06)	0.02	[4.48, 4.72]
	Wall volume (10 × cm^3^)	9.35	0.26 (0.28)	0.03	[8.84, 9.86]

a Gaussian random fields with a standard deviation *σ*_KL_  =  0.5 radians and correlation length *l* equals to 3, 5, and 10 cm. Standard deviation extracted from QMC simulations is also included (QMC).

### 3.2 Impact of input variable uncertainty

**Figure 3:**
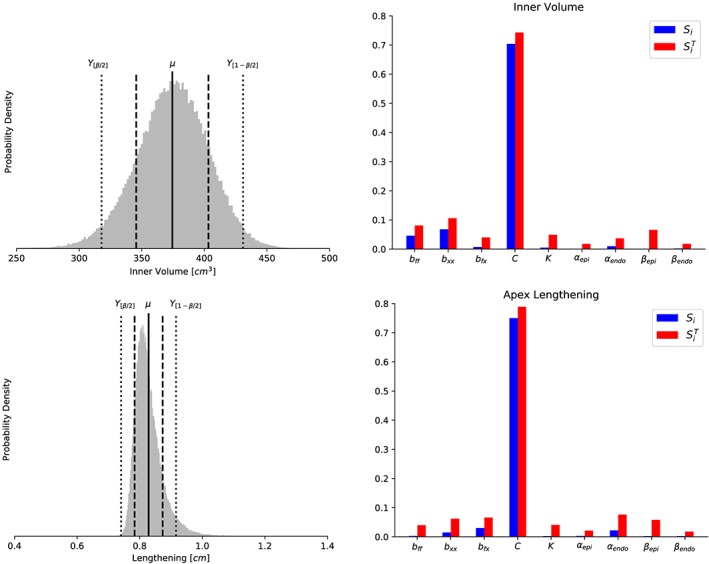
Model A: Probability density (left column) of the inner cavity volume (top) and apex lengthening (bottom) obtained assuming material input parameters included in Table 1. In vertical bars, the mean (solid line), mean ± standard deviation (dashed lines), and the limits of the 95% prediction interval (dotted lines) are shown. Main Sobol index (S_i_, blue) and total Sobol index ( $S_{i}^{T}$, red) are depicted in right column for all quantities of interest

**Figure 4:**
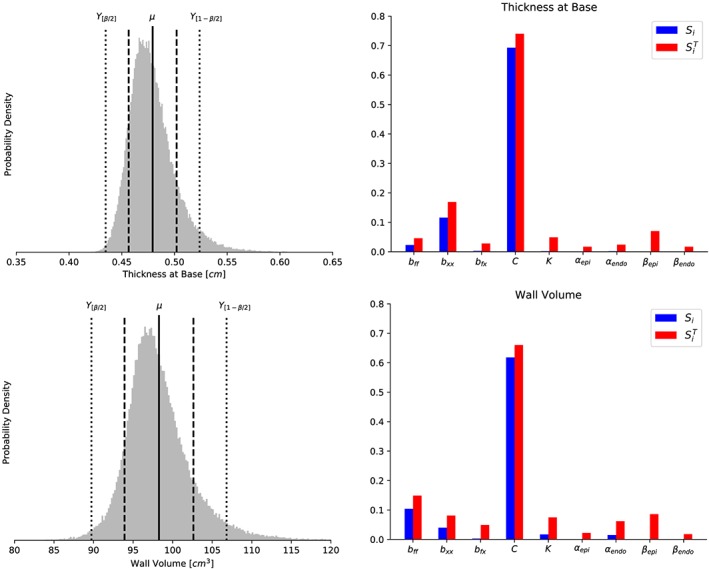
Model A: Probability density (left column) of the wall thickness at base (top) and wall volume (bottom) obtained assuming material input parameters included in Table 1. In vertical bars, the mean (solid line), mean ± standard deviation (dashed lines), and the limits of the 95% prediction interval (dotted lines) are shown. Main Sobol index (S_i_, blue) and total Sobol index ( $S_{i}^{T}$, red) are depicted in the right columns for all quantities of interest

We first consider a UQ analysis of model A, in particular, of the nine model input random variables listed in Table 1 and the four output quantities of interest listed in Table 2. We computed statistical properties of the probability density functions associated with these output quantities, including mean value *μ*, standard deviation *σ*, coefficient of variation *σ*/*μ*, and the 95*%* prediction interval for each output quantity. The resulting statistical quantities are listed in Table 5, and the output density functions are depicted in Figures 3 and 4 (left panels). We observe that all coefficients of variation are at or below 0.08, with the largest coefficient of variation associated with the inner cavity volume and the smallest with the wall volume.

For verification purposes, we also compared the resulting standard variation values with values obtained using QMC directly (without the use of the surrogate PCE model), also listed in Table 5. We observe that the discrepancy in the standard deviation between the PCE and the QMC simulations are less than 2*%* for all output quantities.

From Figures 3 and 4, we observe that output density distributions display a high degree of symmetry (skewness ∼ 0), although with a certain distortion to the right in the case of the apex lengthening especially, but also for the thickness and wall volume, and slightly negatively skewed data in the case of the inner cavity volume.

In addition to the statistical properties reported in Table 5, we computed the main and total Sobol indices with respect to the input random variables for each output quantity. The Sobol indices were only computed using the PCE method, but for verification purposes, we ran a subset of the computations with increased polynomial order (*N*  =  3). This increase did not change the results, leading us to conclude that using quadratic polynomials is sufficient also for calculating the Sobol indices. The indices are plotted in Figures 3 and 4 (right panels) in conjunction with the respective output quantities. Clearly, the uncertainty in the multiplicative factor *C* has the highest main sensitivity index *S*_*C*_ for all four output quantities, with *S*_*c*_  ≥  0.6 for all four cases. More precisely, these sensitivity indices indicate that if *C* was known and fixed to its true value, then the uncertainty in the four output quantities *Q*_*c*_,*Q*_*l*_,*Q*_*t*_, and *Q*_*v*_ would be reduced by 70*%*, 75*%*, 69*%*, and 61*%*, respectively.

For the inner cavity volume, wall thickness, and wall volume, the material stiffness parameters *b*_*ff*_ and *b*_*xx*_ have main sensitivity index in the range 0.02 to 0.15, with *b*_*xx*_ having higher index than *b*_*ff*_ in the case of thickness at base, while *b*_*ff*_ have higher index than *b*_*xx*_ in the case of wall volume and inner cavity volume. In particular, *b*_*xx*_ yields a main sensitivity index greater than 0.05 in the cases of inner cavity volume and thickness and thus emerges as a key parameter for these output quantities. Similarly, *b*_*ff*_ emerges as a key parameter for the wall volume. For these output quantities, the main sensitivity index for the other variables (*b*_*fx*_, *K*, *α*_*epi*_, *α*_*endo*_, *β*_*epi*_, *β*_*endo*_) essentially vanish. For the apex lengthening, we observe that the main sensitivity indices associated with *b*_*fx*_, *b*_*xx*_, and *α*_*endo*_ are small but non‐vanishing, while the main sensitivity index associated with other variables (*b*_*ff*_, *K*, *α*_*epi*_, *β*_*epi*_, *β*_*endo*_) essentially vanishes. Overall, the direct contributions of the angles *α*_*epi*_, *β*_*endo*_, and *β*_*epi*_ to the total variance of any output of interest are negligible: The main sensitivity index of all three angles is close to zero for for all the model outputs.

The total‐order sensitivity indices identify the input variables that may be fixed over their range of variability without affecting some specific output variance. Indeed, these are those inputs corresponding to $S_{i}^{T}≃0$ for all the quantities of interest.^9^, ^10^ We observe that the angles *α*_*epi*_ and *β*_*endo*_ satisfy this condition. These input parameters have the smallest total sensitivity indices for all the output quantities, and in particular, these inputs could be fixed in subsequent model calibrations within their range of uncertainty introducing only about 2*%* of the current output variances. Finally, the total and main sensitivity indices are of similar value for all input parameters, which indicates no significant high‐order interaction between the model inputs.

### 3.3 Impact of fiber field uncertainty

**Figure 5:**
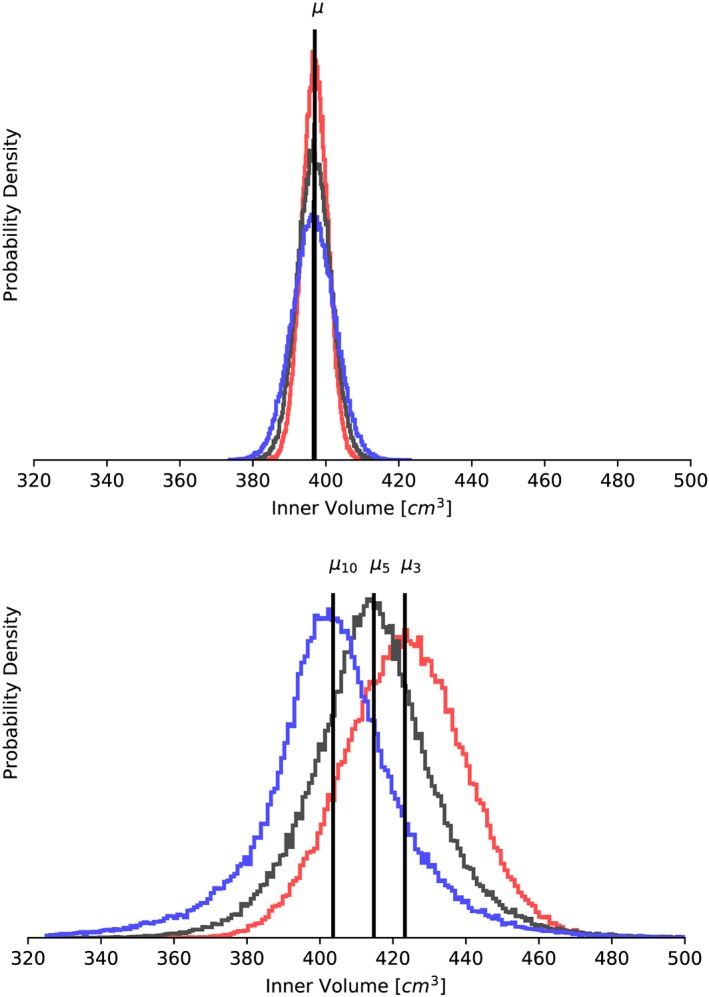
Model B: Probability density functions for the inner volume cavity of the left ventricle assuming two Gaussian random fields as uncertainty in the myofiber architecture: (top) σ_KL_  =  0.1 radians, (bottom) σ_KL_  =  0.5 radians. Colors correspond to different correlation lengths: red (l  =  3 cm), dark gray (l  =  5 cm), and light gray (l  =  10 cm)

**Figure 6:**
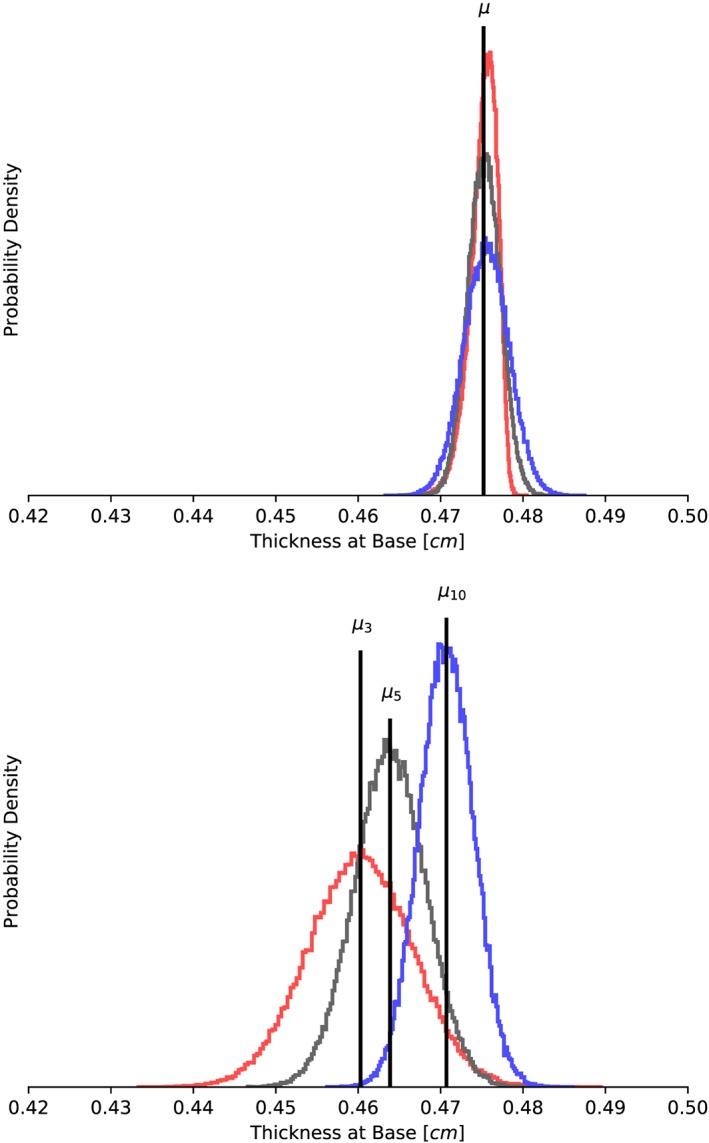
Model B: Probability density functions for the thickness at base assuming two Gaussian random fields as uncertainty in the myofiber architecture: (top) σ_KL_  =  0.1 radians, (bottom) σ_KL_  =  0.5 radians. Colors correspond to different correlation lengths: red (l  =  3 cm), dark gray (l  =  5 cm), and light gray (l  =  10 cm)

We next turn to consider a UQ analysis of model B, where the local fiber orientation is modeled as a Gaussian random field. We consider a total of six cases, combining standard deviations *σ*_KL_ of 6.0° (0.1 radians) and 28.6° (0.5 radians) with correlation lengths 3, 5, and 10 cm. The chosen standard deviation values are based on fiber angle variabilities reported in the literature, which include either measurement errors or a combination of measurement error and biological variations. Samples of the resulting random fields are illustrated in Figure 2. For all six cases, we computed statistical properties of the probability density functions associated with these global response quantities, including the mean value *μ*, standard deviation *σ*, coefficient of variation *σ*/*μ*, and the 95*%* prediction interval for each output quantity listed in Table 2. Statistical measures are presented in Tables 6 and 7, while the probability density functions are illustrated in Figures 5 and 6 for the inner volume and the wall thickness, as most relevant output quantities of interest.

For verification purposes, we also compared the resulting standard variation values with values obtained using QMC directly (without the use of the surrogate PCE model), also listed in Tables 6 and 7. We observe that the discrepancy in the standard deviation between the PCE and the QMC simulations is small, typically of the order 1% to 3% for the range of output quantities and perturbation fields examined.

Table 6 shows the results of modeling the fiber orientation as a Gaussian random field with a standard deviation of 6° (0.1 radians). We see that all quantities of interest show a constant mean value, independent of the correlation length. The coefficients of variation decreases slightly with decreasing correlation lengths for all quantities of interest except the inner volume, for which its coefficient of variation stays constant at 0.01 across the correlation lengths investigated. Different and more interesting patterns are observed when the standard deviation is increased to 0.5 radians, as shown in Table 7. In this case, cavity volume increases from 403 to 424 (cm^3^) as the correlation length decreases from 10 to 3 (cm). The standard deviation stays approximately constant, and the coefficient of variation is 0.04 for all correlation lengths. Turning now to the apex lengthening, we observe that this is the quantity of interest with the largest coefficient of variation, at 0.07 to 0.08 for the correlation lengths examined, with slightly increasing coefficient of variation with increasing correlation length (Table 7). We also observe a slight decrease of the expected value with less correlation in the myofiber variability. Figure 5 shows density distributions for the cavity volume obtained with different perturbation fields. It can be seen that the degree of symmetry remains the same across the correlation lengths compared for both random fields under study. From kurtosis and skewness values (not shown), we confirm that the distributions associated to the inner volume, for both *σ*_KL_  =  0.1 and 0.5 radians, can be considered as univariate normal distributions (absolute value of both skewness and kurtosis are within the range ±1.96^77^, ^78^). However, in the case of *σ*_KL_  =  0.5 radians, as the correlation length *l* increases, the distributions have heavier tails and sharper peak than the normal distribution, while as the correlation of the fiber noise decreases, the diastolic volume results are closer to a Gaussian curve.

From Table 7, we observe that the expected values of both the wall volume and wall thickness decrease with decreasing correlation length, ie, as the uncertainty in the fiber field approaches a *white noise* field. The opposite trend holds in terms of the spread or relative uncertainty for these two response quantities; the coefficient of variation increases with increasing correlation length, although always below 3*%*. These findings are also contrary to the results mentioned above for a narrower width of the perturbation, for which the coefficient of variation diminishes as *l* decreases. Additionally, the skewness and kurtosis values for those two response quantities are close to zero (absolute value of both moments are within the range ±1.96 77, 78), and thus, wall volume and wall thickness distributions fit normal curves, as is also illustrated by Figure 6 for the former quantity. It is interesting to note that in the particular case of correlation length *l*  =  3 cm and *σ*_KL_  =  0.1 radians, we observe slightly negatively skewed data, with the left tail of the density distribution being longer and its mass concentrated on the right of the figure.

## 4 DISCUSSION

The aim of this paper has been to analyze a computational model describing the passive filling phase of the left ventricle using the framework of uncertainty quantification. Our study quantifies the impact of uncertainty in global material parameters, and, more importantly, in measurements of local fiber orientations. The equations governing the passive mechanical behavior of the heart have been solved using the finite‐element software FEniCS,^74^ and the implemented uncertainty framework is based on polynomial chaos expansions accessible via ChaosPy^75^ and truncated Karhunen‐Loéve expansions. This nonintrusive method allowed a successful study of the impact of uncertainties, providing statistical analysis through the probability densities of a set of global response model outputs (inner cavity volume, apex lengthening, wall thickening, and wall volume).

In our first simulation model, we identified the main uncertain input parameters and characterized these by random variables obeying certain specific probability distributions. The results clearly point at the multiplicative factor *C* as the parameter with the largest influence on the variance in model outputs, and *K*, *α*_*epi*_, *β*_*endo*_, and *β*_*epi*_ as the inputs with the lowest impact on model response uncertainty. Furthermore, the SA results indicate that uncertainty in *C* may account for up to 75*%* of the uncertainty in the considered output quantities. Our results suggest that the directional material stiffnesses, both in fiber and cross‐fiber directions, contribute less to overall model output variance, but that these parameters are important for wall volume and to some extent wall thickness. On the other hand, our results from model A indicate that randomness in all angle variables, except to some extent the angle at the endocardiac surface (*α*_*endo*_), contribute very little to the variance of all output quantities of interest. Thus, even rather rough estimates of these parameters would have little effect on the uncertainty in the output predictions. The significance of the *C* parameter is not unexpected, as this parameter directly scales the stress‐strain response and thereby the stiffness of the tissue. However, it is somewhat less intuitive that the sensitivity of *C* is so much larger than the exponent factors *b*_*ff*_,*b*_*xx*_, and *b*_*fx*_. The low sensitivity of the fiber angle parameters can partially be explained by the relatively low degree of anisotropy in our model, although the results from model B indicate that anisotropy still plays a role. The findings are largely in agreement with previous results in Osnes and Sundnes,^29^ which considered the influence of uncertainty in material parameters in a similar model of ventricular mechanics. However, the study in Osnes and Sundnes^29^ considered an even simpler LV model, with idealized and perfectly symmetric geometry, and a perfect match between the results should therefore not be expected.

Modelling uncertainty in the input parameters for the LDRB algorithm examines only one aspect of the influence of randomness in myocardial fiber architecture. For a more thorough study, we therefore also considered Gaussian perturbation fields of a base LDRB‐generated fiber orientation field, thus introducing local perturbations in fiber angle orientation over the computational geometry. Our results reveal that for moderate variability in fiber fields (*σ*_KL_  =  0.1 radians), the impact on all output quantities of interest is fairly low. The mean values stay constant independent of the correlation length of the field, and the coefficients of variation are small and decrease slightly with decreasing correlation length. For larger field variability (*σ*_KL_  =  0.5 radians), the influence of the correlation in myofiber uncertainty differs depending on the quantity of interest; for the inner cavity volume, the relative uncertainty does not change with the correlation length of the perturbation field, while wall properties, such as thickness or wall volume, experience a larger variation relative to the mean as the correlation length decreases. In contrast, our findings demonstrate the opposite behavior for the lengthening variation of the left ventricle at apex. Moreover, our results indicate that the variability of the cardiac tissue in terms of fiber arrangement has a greater influence on apex lengthening (coefficient of variation up to 0.08) than any of the parameters considered in model A (coefficient of variation 0.06). This is the only quantity of interest where this is observed. Both for the apex lengthening and inner cavity volume we found nonnegligible coefficients of variation for the variability in fiber orientation, independent of the correlation length.

The apparent discrepancies between models A and B have some interesting implications. While most model outputs showed a very low sensitivity to the global input parameters of the LDRB algorithm, the experiment with large local variations in fiber orientation showed a large impact on the output quantities. These results indicate that as long as a structured, helical arrangements of fiber orientations is maintained, the precise angles of rotation are not that important. On the other hand, any loss of the helical structure, which is seen in model B for low correlation lengths (Figure 2 right panel), has a substantial impact on the global mechanical properties of the ventricle. In real‐world applications, including patient‐specific simulations, the use of rule‐based assignment of fiber orientation will therefore tend to exaggerate tissue organization and thereby the ventricular stiffness. On the other hand, DTMRI‐based fiber fields capture both true tissue variations and measurement noise and are likely to underestimate the inherent stiffness of the ventricle.

Future studies may target some of the limitations in this work as discussed here. First, the input parameter uncertainty was modelled using prespecified normal/log‐normal type distributions, which were all assumed to be independent. For an even more realistic UQ analysis, one should calibrate these probability distributions in accordance with physiological or medical data (if available) via, eg, Bayesian inversion. One likely result of such analysis is that the input parameters are not completely independent. However, given the limited knowledge on the material properties used in heart mechanics, and the wide range of values used in the literature, the assumption of independent and prescribed probability distributions appears to be a reasonable starting point. Second, in order to quantify the variability of the fiber architecture, while we here considered a truncated Karhunen‐Loéve expansion, an alternative would be a principal component analysis (PCA). PCA may offer a more realistic quantification of the variability of the fiber perturbation field by its mean and covariance matrix sampled from a cardiac diffusion tensor imaging (DTI) population distribution.^28^ Third, other extensions of this study should include not just the filling phase of the heart but also the active contraction of the muscle in the cardiac cycle, as well as taking into account in the same model the uncertainty emerging from both input material parameters and the fiber architecture. On the methods side, the use of a fixed PCE basis may lead to unaffordable computational loads as the number of uncertain parameters grows. For the moderate numbers considered here, the fixed basis proved sufficient, but it may become a limitation for more complex such as bi‐ventricular models with noisy fiber fields. For these cases, adaptive methods should be considered; see, for instance, previous studies.^46^, ^47^, ^48^, ^49^, ^50^ Although computational efficiency was not a topic of focus in our study, UQ on more complex models may lead to challenging computational problems, and performing an efficiency‐oriented survey of UQ methods and method choices would be valuable for future use in the cardiac modeling field.

Finally, an important limitation of the present study is that we only consider the propagation of model parameter uncertainty through a forward model of passive cardiac mechanics and only consider a limited set of output parameters. This choice was made to limit the number of input and output variables, to facilitate the analysis and interpretation of the results. Future work may extend the model to consider the full cardiac cycle and to consider other and more case‐specific output quantities. Another point worth noting is that in typical applications of cardiac mechanics models, material parameters such as *C*,*b*_*ff*_,*b*_*xx*_, and *b*_*fx*_ are fitted to match data from patient recordings or experiments. In this context, the quantities considered as output variables in the present study become input to a parameter estimation problem.^44^ The results obtained in the present study are valuable also in this context, since input variables with high sensitivity indices will be most easily identifiable in an inverse problem setting, while the variables with low sensitivity are essentially non‐observable. However, performing a proper UQ of this parameter estimation problem, quantifying how measurement error impact estimated parameters and in turn model predictions, will be a highly relevant extension of the present work.

## 5 CONCLUSION

We have performed a detailed UQ and sensitivity analysis of a computational model of passive ventricular mechanics, using a PCE method in combination with a Karhunen‐Loéve expansion of stochastic field variables. The methods were verified by comparing selected outputs with results of quasi‐Monte Carlo simulations, confirming that the PCE approach gives an accurate and computationally efficient representation of uncertainty propagation through the cardiac mechanics model. The UQ and sensitivity analysis can be concluded in two main findings. The first is that the the multiplicative factor that scales the strain energy (*C*) is the most sensitive parameter in the material law considered here. The second is that while all considered model outputs are relatively insensitive to the global endocardial and epicardial helix angles, they are highly sensitive to local variations and noise in the fiber orientation.

## CONFLICT OF INTEREST

The authors confirm that there are no conflicts of interest related to this publication.
